# Pesticide exposure of workers in apple growing in France

**DOI:** 10.1007/s00420-021-01810-y

**Published:** 2021-11-10

**Authors:** Mathilde Bureau, Béatrix Béziat, Geoffroy Duporté, Valérie Bouchart, Yannick Lecluse, Emmanuelle Barron, Alain Garrigou, Marie-Hélène Dévier, Hélène Budzinski, Pierre Lebailly, Isabelle Baldi

**Affiliations:** 1 BPH Center, Inserm U1219, Université de Bordeaux, ISPED, Case 11, 146 rue Léo Saignat, 33076 Bordeaux Cedex, France; 2 Univ. Bordeaux, CNRS, UMR5805 EPOC-LPTC, 351 cours de la Libération, 33400 Talence, France; 3 LABÉO, Saint Contest, 1 route de Rosel, 14000 Caen, France; 4 Normandie Univ, UNICAEN, INSERM, UMR 1086 ANTICIPE, 14000 Caen, France; 5 Centre de Lutte Contre Le Cancer François Baclesse, 3 avenue du Général Harris, 14000 Caen, France; 6 CHU de Bordeaux, Service Santé Travail Environnement, Place Amélie Raba Léon, 33000 Bordeaux, France

**Keywords:** Pesticides, Occupational exposure, Dermal contamination, Apple growing, Captan, Dithianon

## Abstract

**Objective:**

Although apple trees are heavily sprayed, few studies have assessed the pesticide exposure of operators and workers in apple orchards. However, these data are crucial for assessing the health impact of such exposures. The aim of this study was to measure pesticide exposure in apple growing according to tasks and body parts.

**Methods:**

A non-controlled field study was conducted in apple orchards in 4 regions of France during the 2016 and 2017 treatment seasons. Workers’ external contamination and their determinants were assessed over 156 working days corresponding to 30 treatment days, 68 re-entry days and 58 harvesting days. We measured pesticide dermal contamination during each task and made detailed observations of work characteristics throughout the day. Captan and dithianon were used as markers of exposure.

**Results:**

The median dermal contamination per day was 5.50 mg of captan and 3.33 mg of dithianon for operators, 24.39 mg of captan and 1.84 mg of dithianon for re-entry workers, and 5.82 mg of captan and 0.74 mg of dithianon for harvesters. Thus, workers performing re-entry tasks, especially thinning and anti-hail net opening, presented higher contamination, either equal to or higher than in operators. For these last ones, mixing/loading and equipment cleaning were the most contaminating tasks. Most of the contamination was observed on workers’ hands in all tasks, except for net-opening in which their heads accounted for the most daily contamination.

**Conclusions:**

This study highlights the importance of taking indirect exposures into account during re-entry work in apple growing.

## Introduction

Numerous epidemiological studies have found associations between occupational pesticide exposure and health issues such as cancers, neurological diseases and reproductive disorders (Blair et al. 1992; Acquavella et al. 1998; Colosio et al. 2003; Alavanja et al. 2004). However, pesticide exposure assessment remains a critical issue and exposure levels in real working conditions are poorly known. Some studies provide data on the pesticide exposure of operators in open-field crops (Wojeck et al. 1983; Abbott et al. 1987; Hines et al. 2001; Lebailly et al. 2009; Aprea et al. 2016), vineyards (Wojeck et al. 1982; Baldi et al. 2006; Fustinoni et al. 2014), fruit growing (Hansen et al. 1978; Karr et al. 1992; Hines et al. 2011; Moon et al. 2013; Kim et al. 2015; Lee et al. 2018) and greenhouses (Adamis et al. 1985; Fenske et al. 1987; Machera et al. 2003). These studies have assessed pesticide dermal contamination, respiratory exposure and biological levels, thereby helping to develop tools to estimate pesticide exposure among operators. In addition, they have enabled an assessment of levels of contamination according to specific tasks and, in some, an identification of the major determinants of exposure. They have also offered a better understanding of the usual conditions of work in non-controlled conditions. Numerous studies have suggested that the dermal route greatly contributes to pesticide exposure during occupational outdoor tasks, but contamination may also occur through the respiratory route, especially when working with highly volatile pesticides or in confined spaces (Dowling and Seiber 2002).

Few studies have documented indirect pesticide exposure of workers, for instance during re-entry tasks in fruit growing (peaches, apples, citrus fruits). Six studies on exposure in apple growing have been conducted in the United States (Wolfe et al. 1975; Davis et al. 1982, 1983; Fenske et al. 1999, 2003; Simcox et al. 1999), while two were carried out in the Netherlands (de Cock et al. 1998b; Tielemans et al. 1999). In general, pesticide exposure is assessed a few hours or days after the last application during thinning, considered as a contaminant task. Exposures during harvesting, bending and pruning have been assessed more rarely.

In the studies on exposure in fruit growing, dermal contamination was usually measured with skin patches applied on or under clothing. Patches were applied on about ten locations (arms, chest, back, legs, head) to be representative of the whole body (Wolfe et al. 1975; de Cock et al. 1998b; Hines et al. 2011; Moon et al. 2013; Kim et al. 2015) or only applied on upper parts (Davis et al. 1982, 1983). Hand exposure was usually assessed by absorbent gloves, more rarely by hand rinsing (de Cock et al. 1998b; Fenske et al. 1999). The whole body dosimetry method, with inner and outer clothing, was rarely used (McCurdy et al. 1994; Lee et al. 2018). Additionally, an assessment of respiratory exposure was sometimes performed with the use of personal air sampling devices (Wolfe et al. 1975; Davis et al. 1982; de Cock et al. 1998b; Moon et al. 2013; Kim et al. 2015; Lee et al. 2018). In few studies, the internal contamination was monitored through urinary metabolites (Wolfe et al. 1975; McCurdy et al. 1994; Simcox et al. 1999; Fenske et al. 2003) and blood cholinesterase activity (Wolfe et al. 1975; Popendorf et al. 1979).

Apples are among the largest fruit productions worldwide with about 87 million tons per year, either consumed as fresh fruit or transformed (FAO 2019). France ranks third in Europe with 1.7 million tons produced per year, and fruit growing is the third-biggest French agricultural production, after open-field crops and vineyards. In 2019, employment in farms only devoted to fruit growing corresponded to about 38,000 annual work unit (AWU), namely 6% of the French total employment in agriculture (684,000 AWU)(Agreste 2020). In 2010, 27,000 farms, 33,000 farm-owners, 12,000 employees and 15,000 active family members were involved in fruit growing, along with a large seasonal workforce (32% of all annual work on fruit-growing farms)(Agreste 2013). Although only 6% of all French farms produce fruit, they represent 9% of the French agricultural workforce and 27% of the seasonal workforce. This large workforce performs various re-entry tasks in the orchards such as thinning, pruning and anti-hail net management during the pesticide spraying season. Many pesticide applications (up to 40) are performed between March and late August every year in apple orchards. Half of them involve fungicides against *Venturia inaequalis*, which is responsible for apple scab disease, causing significant economic losses.

In the early 2000s, the PESTEXPO program in France was set up in order to assess pesticide exposure with various crops for epidemiological purposes. Levels and determinants of exposure of workers were determined with open-field crops and vineyards (Lebailly et al. 2009; Baldi et al. 2006, 2012, 2014a). The CANEPA (CANcer and Exposure to Agricultural Pesticides) study within the PESTEXPO program aimed to measure and compare levels of dermal contamination in various tasks performed in apple-growing orchards: pesticide treatment, re-entry tasks, and harvesting.

## Materials and methods

The field part of the study took place during the 2016 and 2017 seasons (from March to December) in four areas of France: Normandy, Poitou–Limousin, Garonne Valley and Rhône-Alpes.

Apple-growers were identified with the help of local agricultural organizations (Chambres d’agriculture, FREDON) and cooperatives, or contacted from the phone book. They were selected on the basis of having planned treatments with captan or dithianon in the following season. We provided detailed and standardized information to the participants. Informed consent was obtained from each subject prior to the beginning of the study including authorization to take pictures on the observation day.

In order to extrapolate the results to other active ingredients in future epidemiological studies, two fungicides widely employed against the main fungus diseases, captan and dithianon, were used as markers of external exposure. Captan and dithianon have been widely used in France since 1954 and 1966, respectively. In 2016, 271 tons of captan and 107 tons of dithianon were sold in France (Institut national de l'environnement industriel et des risques (INERIS) (2016)). The commercial products used by the farmers involved in this study were formulated as water dispersible granules (WG) or suspension concentrates (SC): Sigma DG^®^ (concentration: 80%), Merpan^®^ (480 g/l), Merpan 80 WDG^®^ (80%) and Brocelian^®^ (600 g/kg) contained captan; Delan^®^ (70%), Delan WG^®^ (70%), Delan Pro^®^ (125 g/l) and Maccani^®^ (120 g/kg) contained dithianon. In France, the legal re-entry interval is 48 h for these pesticides and they should not be applied less than 24 days before harvesting for captan and 14 days for dithianon.

### Dermal contamination

Dermal contamination was assessed by quantifying the quantities of pesticides in patches placed on the skin, following the Organization for Economic Co-operation and Development (OECD) guidelines (OECD 2002). Hand exposure was assessed using cotton gloves or by handwashing (*N* = 29) when gloves were considered to disrupt the work (discomfort, rainy days).

**Fig. 1:**
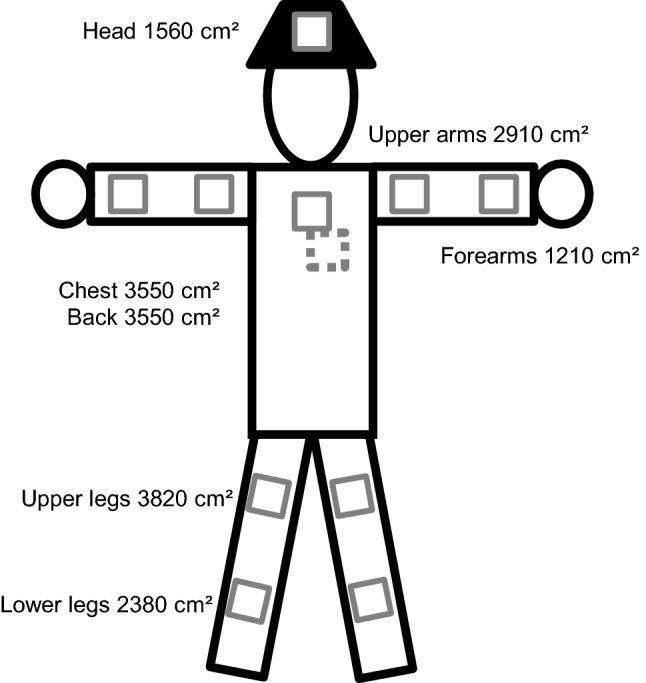
Location of patches and surface of the sampled body part

The patches were made from 10 cm × 10 cm layers of surgical cotton gauze (Méfra^®^) backed with an aluminum foil and a medical adhesive strip (Hypafix^®^). They were placed at 11 locations on the skin of the worker (Fig. 1): forearms (2), upper arms (2), chest, back (between shoulder blades), thighs (2), lower legs (2) and head (patch on the front of a cap). During treatment, the patches were affixed before each phase, i.e., each mixing/loading, spraying and equipment cleaning task. The patches were removed after each phase, individually identified, packed in aluminum foil inside a plastic bag, stored in a cool box before and during transport to the laboratory, and finally transferred to the deep freeze (− 20 °C). During re-entry and harvest days, one set of patches corresponded to a half day of work: the patches were removed at midday, following the steps described previously, and other patches were placed on the skin of the workers after they had had lunch. The patches were not changed when workers worked only a half day, which was especially the case when they started very early in the morning due to high temperatures and did not have a lunch break. If the patches looked likely to fall off during the observation, they were removed and kept for analysis following the steps presented above, and replaced by new ones (17% of all observations).

**Fig. 2:**
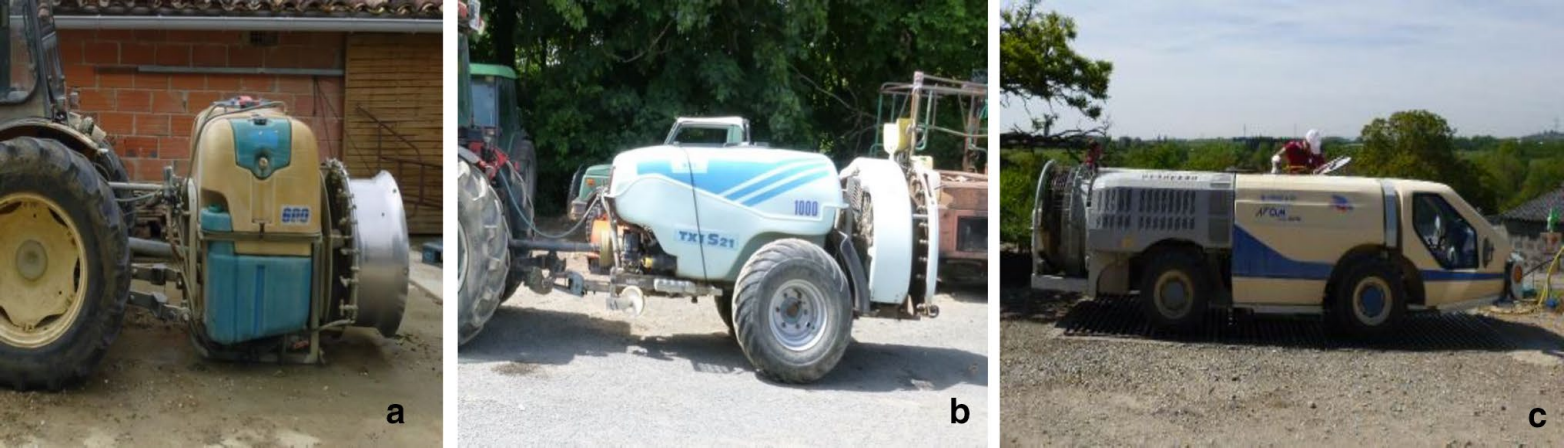
Pictures of the different types of sprayers observed in apple orchards: (**a**) Rear-mounted sprayer. (**b**) Trailed sprayer. (**c**) Self-propelled sprayer

Gloves were put on and removed following the same standardized process. For handwashing, 500 ml of mineral water was poured slowly over the hands of the workers while they rubbed their hands together. The wash water was collected in a disposable aluminium tray, poured into an aluminum bottle and stored like the other samples. Hands were washed before starting work (blank) and after each phase (treatment) or each half-day (re-entry or harvesting tasks).

In 6 observations, the patches and gloves were not removed between mixing/loading and spraying. Therefore, the associated contamination values were considered in the analyses per day of treatment but not per phase, as they could not be distinguished.

The total daily dermal exposure of the workers was calculated from the measured pesticide concentrations on the patches and gloves or handwashing samples. For each body area, a coefficient corresponding to the estimated area of skin in cm^2^ was applied, as recommended by the OECD (Fig. 1). The daily dermal exposure (in mg) was calculated as the sum of all body parts. As captan is highly prone to degradation due to different conditions into tetrahydrophthalimide (THPI)(FAO and WHO 1995), the quantification of captan was performed together with its main metabolite. We calculated captan values as the sum of captan and THPI. On treatment days, we considered contaminations associated with the active ingredient handled on the day of the observation. For re-entry tasks, we considered contamination by both captan and dithianon. The contamination per hour was calculated for each observation as the daily dermal exposure divided by the actual working time.

### Inhalation

During 18 treatment observations, potential inhalation exposure was also investigated by measuring the concentration of compounds in the operator’s breathing zones. No standard method is defined for measuring inhalation contamination by captan, THPI and dithianon. We decided to use XAD-2 filters (OVS-2 tubes), as recommended by National Institute for Occupational Safety and Health (NIOSH 2016). A filter was held to the farmer’s shoulder and connected to a personal air sampling pump (GilAir^®^ Plus, Sensidyne^®^), with a sampling rate of 1 l/min. The filter was changed after each task (mixing/loading, spraying, cleaning), individually packed in aluminum foil, stored in a cool box before and during transport and transferred to deep freeze until analysis. The inhalation dose was calculated from the concentration in the air with a lung ventilation of 28 l/min (U.S. EPA 2011). The inhalation doses of all the phases performed during the observation day were summed to obtain the daily respiratory contamination of the operator.

### Observation days

When they planned a treatment with captan or dithianon, the farmers called the research team the day before. Re-entry and harvesting tasks were planned about one week beforehand. Field monitors attended all tasks during the working day and were instructed to disturb the work as little as possible. They collected variables in standard field notebooks, one for treatment observation and one for re-entry and harvest days. Five categories of variables were collected in both notebooks: (i) characteristics of the farm (farm and orchard areas, other crops, etc.); (ii) characteristics of the worker (name, date of birth, gender, education, status, experience in apple growing and in pesticide application, clothing, personal protective equipment, etc.); (iii) characteristics of the orchard (type, cultivars, height of trees, distance between rows, plot area, etc.); (iv) weather (temperature, humidity, wind speed); and v) workers’ perceptions of their work and exposure. In addition, the task was described in detail (duration, tools, equipment, number of breaks and their duration, explanations about the task and its organization, incidents, etc.). For the treatment observations, the characteristics of the spraying equipment (age of tractor and sprayer, type of sprayer, tank volume, number of nozzles, distance between the seat and the nozzles, etc.) were recorded along with the detailed steps for each mixing, spraying and cleaning phase (commercial products and their quantity, type of containers, spraying speed, volume of mixture per hectare, cleaning of the sprayer, etc.). In addition, the observer took pictures and recorded several videos for ergonomic purposes.

### Analytical methods

Certified analytical-standard captan, THPI and dithianon were obtained from Dr Ehrenstorfer (Augsburg, Germany). Stock solutions at 1000 mg/l were prepared in acetonitrile (ACN) (Biosolve Chimie, Dieuze, France) and standard solutions in ACN/Milli-Q ultrapure water (Merck Millipore, Billerca, MA, USA), 20/80 v/v acidified with formic acid (FA) (Sigma-Aldrich, Bellefonte, PA, USA) 0.1%.

Handwashing waters, gloves and patches were analyzed by high-performance liquid chromatography (HPLC) with a 1100 diode array detector (UV-DAD) from Agilent Technologies (Santa Clara, USA). Dithianon, captan and THPI were extracted from the gloves and patches with respectively 80 ml and 40 ml ACN acidified with 0.1% FA. The vials were shaken for 15 min with STR4 rotator drive (Stuart, Staffordshire, UK.). After recovery, 100 µl of this extract was diluted with 400 µl of ultrapure water acidified to 0.1% FA before injection into the HPLC system. For the handwashing water, 400 µl of acidified 0.1% FA water sample was added to 100 µl ACN before HPLC analysis.

The analytical column was a Nucleoshell RP 18plus, 150 × 4.6 mm, 2.7 µm (Macherey–Nagel, Düren, Germany) kept at 40 °C during analysis. The initial mobile phase was 1% (v/v) ACN and MilliQ ultrapure water, each acidified at 0.1% H3PO4, increased to 50% ACN over 5 min, then 95% over 20 min and held for 7 min. The mobile flow rate was 0.4 ml/min and the volume injected was 100 µl for each sample or standard. The detection wavelength ranged from 190 to 370 nm to take the UV spectra and the quantification wavelength for captan, THPI and dithianon was 200 nm.

Captan, dithianon and THPI were identified according to retention time and correlation with standard UV spectra, then quantified with a calibration table for the concentration ranging from 10 to 2000 µg/l for each compound, using Chemstation software. When the concentration value was above the range of calibration, the extract was diluted with ACN/ultrapure water 20/80 v/v to reach an accurate value.

The Limits of quantification (LOQ) were as follows: 4 µg/glove, 2 µg/patch and 6 µg/handwashing water. Weekly, laboratory blank were extracted and analyzed following the same procedure to determine any potential contamination during the analytical protocol. No contamination has been highlighted in this work. Overall recoveries for the QC samples were 110 ± 7% for THPI, 98 ± 7% for captan, and 89 ± 8% for dithianon.

The XAD-2 filters were analyzed with LC/MS/MS (dithianon) and GC/MS/MS (captan and THPI) to reach lower LOQs than LC–UV–DAD. The LOQs were 2.5 ng/filter for dithianon and 8 ng/filter for captan and THPI.

### Statistical analysis

Parameters of distribution were calculated to describe the population, the characteristics of the tasks and the exposure values. Student’s *t*-tests were performed on log-transformed daily contamination values to examine the difference between the three types of tasks. Pearson’s chi-squared tests were used for testing associations of tasks and personal protective equipment (PPE). Correlation between respiratory contamination and dermal contamination was estimated by performing linear regression and by calculating Pearson’s correlation coefficient. Differences were considered significant if the *P*-value was < 0.05. Statistical analyses were performed with the STATA software (STATA Corporation, release 15.0).

## Results

### Population (Table 1)

**Table 1:** Characteristics of the observation days

	Treatment (*N* = *30)*	Reentry tasks (*N* = *68)*	Harvest (*N* = *58)*
***Location***			
Normandy	4 (13%)	1 (1%)	10 (17%)
Poitou-Limousin	12 (40%)	34 (50%)	21 (36%)
Garonne Valley	10 (33%)	32 (47%)	27 (47%)
Rhône-Alpes	4 (13%)	2 (3%)	0 (0%)
**Farm area** ^a^ ** in ha (n = 24 farms)**	67.1 (11.0–265)	53.0 (11.0–167)	77.7 (11.0–265.0)
**Apple orchard areaa (ha) (n = 24 farms)**	19.2 (1.6–120.0)	19.3 (2.0–120.0)	20.6 (2.0–120.0)
**Number of participants**	27	58	50
***Gender***			
Men	26 (96%)	34 (49%)	34 (68%)
Women	1 (4%)	24 (41%)	16 (32%)
***Job status***			
Farm owners	16 (59%)	8 (14%)	8 (16%)
Permanent employees	11 (41%)	23 (40%)	14 (28%)
Seasonal workers	0 (0%)	23 (40%)	24 (48%)
Trainees and family members	0 (0%)	4 (7%)	4 (8%)
***Education level***			
Vocational certificate (agriculture)	16 (59%)	13 (22%)	10 (20%)
Any degree in agriculture	11 (41%)	6 (10%)	9 (18%)
***If no agricultural education:***			
Low level		24 (41%)	19 (38%)
Secondary school		7 (12%)	3 (6%)
Any degree		3 (5%)	6 (12%)
Not specified		5 (9%)	3 (6%)
***Period***			
Spring	22 (73%)	28^b^ (41%)	0 (0%)
Summer	8 (27%)	30^c^ (44%)	21 (36%)
Fall	0 (0%)	9^d^ (13%)	37 (64%)
Winter	0 (0%)	1^e^ (1%)	0 (0%)
**Temperature (°C)**	23.1 (7–34.5)	21.3 (-5–40.9)	18.6 (4–42.6)
**Duration of the observation (min)**	165 (40–389)	390 (152–540)	352 (210–503)
***Active ingredient handled (treatment)/previously applied on the orchard (re-entry and harvest)***			
Captan only	17	1	4
Dithianon only	13	0	4
Both		67	54
***Time since previous treatment (in days)***			
Captan	17 (1–70)	30 (2–126)	70 (27–171)
Dithianon	24 (1–90)	52 (3–216)	106 (14–188)

Qualitative variable: number (%)

Quantitative variable: mean (range)

^a^Same farm is counted only once

^b^Anti-hail net opening (*N* = 14), thinning (*N* = 12), other tasks (pruning, bending the branches) (*N* = 2)

^c^Thinning (*N* = 30)

^d^Anti-hail net closing (*N* = 7), other tasks (apple packaging) (*N* = 2)

^e^Other task (pruning) (*N* = 1)

Twenty-four farms were enrolled during the 2016 and 2017 seasons: 4 in Normandy, 5 in Rhône-Alpes, 7 in Poitou-Limousin and 8 in Garonne Valley. Among them, 13 farms were completely devoted to fruit growing, 6 also cultivated other crops (vines, open-field crops or vegetables), and 5 had crops and cattle. The mean total farm area was 67.1 hectares (ha) (11–265) and the mean orchard area 20.5 ha (4.8–120).

One hundred and seven volunteers were observed, 70% of them once, 21% twice and 9% three times or more (with a maximum of 7 observations for one participant). Most of them were men (70%), with a mean age of 41.6 years. Only one operator was a woman, observed once. Women were more represented during re-entry tasks (41%) and harvesting (32%). Operators were 59% farm owners and 41% permanent employees. Farm owners were less observed during re-entry days (14%) and harvesting days (16%). Seasonal workers were frequently observed during re-entry (40%) and harvesting days (48%). Other workers were mostly permanent employees (40% of re-entry workers and 28% of harvesters). All the operators had received some agricultural education, versus only 33% of re-entry workers and 38% of harvesters. The others had mostly low educational levels (primary school or vocational certificate) (41% of re-entry workers and 38% of harvesters). Five re-entry workers (9%) and three harvesters (6%) did not give any information about their educational level.

### Characteristics of the observation days (Table 1)

Overall, 156 observation days were conducted over the two seasons: 69 in 2016 and 87 in 2017, corresponding to 30 treatment days, 68 re-entry days and 58 harvesting days. Observations lasted on average 165 min for treatment, 390 min for re-entry tasks and 352 min for harvesting. Re-entry observations took place on average 30 days (25^th^ percentile: 8) after a captan application and 52 days (25^th^ percentile: 18) after a dithianon application.

### Characteristics of each task (Table 2)

**Table 2:** Main characteristics of each task

Quantitative data Qualitative data	Mean *N*	Range %
**Mixing phases** (*N* = 52)		
*Duration per phase (min)*	14.6	4–47
*Handled active ingredient*		
Captan	34	65
Dithianon	18	35
*Quantity of active ingredient (kg)*		
Captan	5.6	0.9–9.6
Dithianon	1.8	0.5–2.3
*Technical issues*	12	23
**Spraying phases** (*N* = 52)		
*Duration per phase (min)*	78	14–129
*Active ingredient concentration (g/l)*		
Captan	4.4	1.7–8.0
Dithianon	1.2	0.5–1.6
*Treated surface (in ha)*	3.4	0.6–6.5
*Tractor type*		
Four sides closed cabin	49	94
No cabin	3	6
*Sprayer type* (Fig. 2)		
Rear-mounted sprayer	8	15
Self-propelled sprayer	2	4
Trailed sprayer	42	81
*Operator get off the tractor during spraying (yes)*	20	39
**Equipment cleaning phases** (*N* = 12)		
*Duration per phase (min)*	9.9	2–21
**Re-entry tasks** (*N* = 68)		
*Type of task observed*		
Anti-hail net opening	14	21
Manual thinning	42	62
Anti-hail net closing	7	10
Pruning and bending the branches	3	4
Apple packaging	2	3
*The task lasted the whole day (yes)*	65	96
**Harvest days** (*N* = 58)		
*Type of harvesting*		
Manual harvesting	48	83
Mechanical harvesting	10	17
*The task lasted the whole day (yes)*	41	71

#### Mixing operations (*N* = 52)

A single mixing phase lasted 15 min on average (4–47 min). The volume of mixture in the tank averaged 1,305 l (150–3000 l). Captan and dithianon were handled in 34 and 18 mixing phases, respectively, with a mean quantity of 5.6 kg and 1.8 kg per operation, respectively. WG formulations were used during all mixing phases handling captan and during half of mixing operations using dithianon (*N* = 9); otherwise, SC formulations were used. Technical issues occurred in 12 mixing operations (23%): operators were touched by spillages due to mechanical problems (*N* = 3), foaming (*N* = 1) or when pouring the product into the tank (*N* = 8).

#### Spraying operations (*N* = 52)

A spraying task lasted 78 min on average (14–129 min). The mean calculated concentration of captan and dithianon in the mixture was 4.4 g/l (1.7–8 g/l) and 1.2 g/l (0.5–1.6 g/l), respectively. Tractors without cabins were observed in 3 spraying operations; others had a four-side closed cabin. Trailed sprayers were commonly used (94% of spraying phases). During 20 spraying phases, operators were observed getting off the tractor in the field, to unblock a nozzle or open/close additional nozzles (*N* = 9), activate the rinsing tank (*N* = 3), for mechanical problems (*N* = 3) or external events (e.g. removing obstacles, interruption by a colleague) (*N* = 5).

#### Equipment cleaning (*N* = 12)

Equipment cleaning was observed at the end of 6 treatments using captan and 6 using dithianon, and consisted of an external clean of the tractor and the sprayer (*N* = 11) or only an inside clean of the tank (*N* = 1). This operation lasted almost 10 min. No technical issue was observed.

#### Re-entry tasks (*N* = 68)

The re-entry tasks observed corresponded to manual thinning (*N* = 42), anti-hail net opening (*N* = 14) and closing (*N* = 7), and various tasks such as apple packaging (*N* = 2) and pruning or bending the branches (*N* = 3). Performing these tasks took the whole working day in 65 observations (96%).

#### Harvesting days (*N* = 58)

The apples were harvested by hand in 83% of observations. Mechanical harvesting was also observed 10 times in farms located in Normandy, producing apple cider. Harvesting took the whole working day in 41 observations (71%).

### Personal protective equipment (Table 3)

**Table 3:** Personal protective equipment on workers during the various tasks

	Wore gloves^a^ (yes)		Wore a carbon mask (yes)		Wore a coverall^b^ (yes)		Bare forearms^c^		Bare lower legs^c^	
	*N*	%	*N*	%	*N*	%	*N*	%	*N*	%
**Treatment operators**										
Mixing (*N* = 52)	44	85	43	83	21	40	14	27	51	10
Spraying (*N* = 52)	5	10	4	8	7	14	34	65	6	31
Equipment cleaning (*N* = 12)	11	92	4	33	3	25	9	75	5	42
*p*-value^d^	< 0.001		< 0.001		0.01		< 0.001		0.01	
**Re-entry workers** (*N* = 68)	10	17	0	0	0	0	48	71	30	44
Anti-hail net opening (*N* = 14)	6	43	0	0	0	0	8	57	5	36
Thinning (*N* = 42)	2	5	0	0	0	0	35	83	25	60
Anti-hail net closing (*N* = 7)	2	29	0	0	0	0	3	43	0	0
Other (*N* = 5)	0	0	0	0	0	0	2	40	0	0
*p*-value^e^	< 0.001		–		–		0.04		0.12	
**Harvest workers** (*N* = 58)	2	3	0	0	0	0	33	57	10	17
*p*-value^f^	0.03		–		–		0.11		0.001	

^a^Chemical resistant gloves for operators; cut resistant gloves for reentry and harvest workers

^b^Coverall, apron or coat

^c^Bare during some hours or all day long

^d^Differences between Mixing, Spraying and Cleaning (Pearson’s chi-squared test)

^e^Differences between Anti-hail net opening and Thinning (Pearson’s chi-squared test)

^f^Differences between Re-entry and Harvest (Pearson’s chi-squared test)

Most operators wore PPE during mixing/loading operations: chemical resistant gloves in 44 mixing phases (85%) and masks in 43 (83%), but coveralls, aprons and coats were worn less often (40%). Operators rarely wore PPE during spraying operations (15%): gloves in 5 spraying phases (10%), masks in 4 (8%) and coveralls in 7 (14%). Operators in a no-cabin tractor (corresponding to 3 spraying phases) wore gloves, a mask and a coverall. One participant drove an old tractor (20 years) with a four-side cabin without an air-conditioner or filter; he wore a coverall and gloves. All operators except one wore gloves for equipment cleaning (92%), but they wore a mask for only 4 cleaning operations (33%) and a coverall for 3 (25%).

Operators had bare forearms in 14 mixing phases (27%), 34 spraying tasks (65%) and 9 cleaning operations (75%). They had bare lower legs in 5 mixing (10%), 16 spraying (31%) and 5 equipment cleaning tasks (42%).

No re-entry or harvest workers wore a mask or a coverall. Re-entry workers wore cut-resistant gloves in 10 observations (15%), especially during anti-hail net management. They had bare forearms in 48 observations (71%), especially on thinning days when the weather was hot. They had bare lower legs on 30 (44%) re-entry days. Only two (3%) harvest workers wore cut-resistant gloves. A majority had bare forearms (57%). They had bare lower legs on 10 (17%) harvest days.

### Levels of contamination

#### Contamination during the various tasks (Fig. 3a)

**Fig. 3:**
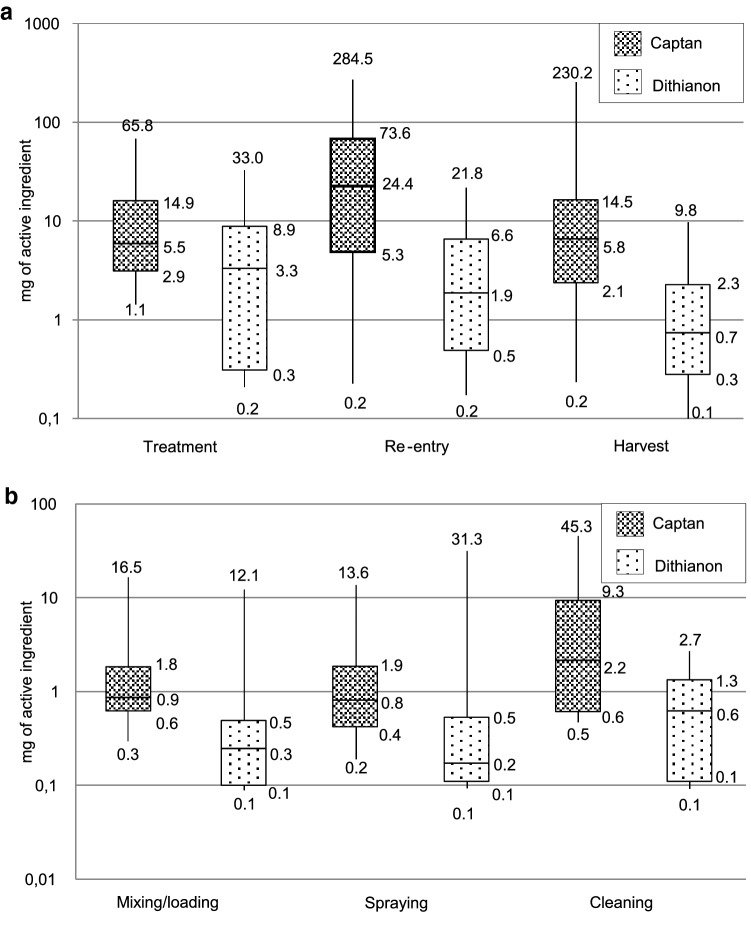
a Distribution of the dermal contamination of workers during treatment (*N* = 17 [captan]; *N* = 13 [dithianon]), re-entry (*N* = 68 [captan]; *N* = 67 [dithianon]) and harvest (*N* = 54 [captan]; *N* = 54 [dithianon]) (minimum, 25e percentile, median, 75e percentile, maximum). b Distribution of the dermal contamination of operators during mixing/loading (*N* = 28 [captan]; *N* = 18 [dithianon]), spraying (*N* = 29 [captan]; *N* = 18 [dithianon]) and cleaning (*N* = 6 [captan]; *N* = 6 [dithianon]) (minimum, 25e percentile, median, 75e percentile, maximum)

The median dermal contamination was 5.50 mg for operators handling captan (*N* = 17) (1.09–65.75 mg) and 3.33 mg (0.19–32.99 mg) for operators handling dithianon (*N* = 13). The median contaminations per hour were 1.90 mg of captan and 0.93 mg of dithianon.

Levels of contamination during re-entry days ranged from 0.23 to 284.49 mg of captan (median: 24.39 mg) and from 0.17 to 21.80 mg of dithianon (median: 1.87 mg). The highest levels of contamination were observed during thinning (*N* = 42) and anti-hail net opening (*N* = 14), with a median of 37.93 mg and 18.29 mg of captan, respectively. During anti-hail net closing, the median contamination was 0.95 mg. The median daily contamination for harvesters was 5.82 mg of captan (0.20–230.16 mg) and 0.74 mg of dithianon (0.08–9.75 mg). The medians contaminations per hour were 4.03 mg of captan and 0.26 mg of dithianon in re-entry workers, and 0.84 mg of captan and 0.08 mg of dithianon in harvesters.

Levels of captan were significantly higher during re-entry days than harvest days (*P* < 0.001) and treatment days (*P* = 0.03). There was no significant difference between contamination during treatment days and harvest days (*P* = 0.25). For dithianon, levels of contamination were also significantly higher during re-entry days than harvest days (*P* = 0.002). Operators were more contaminated than harvesters (*P* = 0.03), but no significant difference was found between operators and re-entry workers (*P* = 0.44).

#### Contamination in operators (Fig. 3b)

Levels of contamination varied according to the treatment phases. For captan, the median dermal contamination was 0.86 mg for mixing/loading (*N* = 28), 0.81 mg for spraying (*N* = 29) and 2.15 mg for equipment cleaning (*N* = 6). For dithianon, the median dermal contamination was 0.25 mg for mixing/loading (*N* = 18), 0.17 mg for spraying (*N* = 18) and 0.62 mg for cleaning operation (*N* = 6). The maximal values of each phase were observed in the same operators.

The average contribution of each phase to the daily dermal contamination was 42% for mixing/loading, 30% for spraying and 33% for cleaning. When no cleaning was performed, mixing/loading contributed the most to dermal exposure in 11 operators, while spraying ranked first in 6 observations.

#### Respiratory contamination

Respiratory contamination was measured over 10 treatment days with captan, corresponding to 14 mixing/loading and 13 spraying phases, and over 7 treatment days with dithianon, corresponding to 7 mixing/loading and 9 spraying phases. The inhalation values of 2 mixing tasks and 1 spraying task were missing due to a technical issue with the pump. In operators handling captan, the daily contamination averaged 0.04 mg of captan (0.02–0.22 mg). In operators handling dithianon, lower values of daily contamination were measured, on average 0.0007 mg (0.0005–0.03 mg). The median respiratory contamination during captan spraying was 0.02 mg (range 0.0003–0.06), twice that during mixing (median 0.01; range 0.0003–0.07). For dithianon too, the median respiratory contamination during spraying (0.0007 mg, range 0.00005–0.01) was higher than that during mixing (median 0.0003 mg; range 0.00005–0.002). The contribution of mixing to respiratory exposure was 36% on average and ranged from 2 and 84%. The mean contribution of spraying was 64%, ranging from 16 to 98%.

For treatment days with dithianon, inhalation values were positively correlated to daily dermal contamination (*r* = 0.99, *P* < 0.001) and to dermal contamination during spraying (*r* = 0.90, *P* < 0.001). However, inhalation values were not correlated to dermal contamination during mixing (*r* = 0.19, *P* = 0.68). For treatment days with captan, inhalation values were not correlated to daily dermal contamination (*r* = 0.17, *P* = 0.65), dermal contamination during mixing (*r* = 0.23, *P* = 0.43) and during spraying (*r* = 0.21, *P* = 0.49).

#### Contribution of each body part in dermal contamination

During treatment days, hands accounted for half of the dermal contamination (49%); for mixing/loading the value was 45%, spraying 39% and cleaning 38%. The lower legs and trunk contributions averaged about 15%–20% each and ranked second and third for each phase and a whole day of treatment. Considering all re-entry days, hands accounted for half of the daily dermal contamination (48%). However, values varied with the type of re-entry tasks performed. Hands contribution was higher during thinning (61%), and lower in anti-hail net management (34%). In anti-hail net opening, the most exposed body part was the head, accounting for 39% of the daily dermal contamination, unlike thinning in which the head accounted for 9%. On harvest days, the hands were the most contaminated part (38%), followed by the forearms, trunk and lower legs (about 15% each).

## Discussion

CANEPA was the first non-controlled study in France on pesticide exposure in fruit growing. It provided original exposure data on a wide range of practices and situations, including indirect exposures, among operators, re-entry and harvest workers in apple orchards. An important finding is that the daily pesticide exposure of re-entry workers appeared to be equal to or higher than the exposure of operators. Thinning and anti-hail net opening were associated with the highest contaminations. Significant levels of dermal contamination were also observed in harvest workers.

When enrolling participants, we strove to ensure diversity in terms of types of farm, equipment and practices in various French regions. Despite these efforts, all farm owners and workers were volunteers and cannot be considered fully representative of French apple-growers. In our study, the mean orchard area (20.5 ha) was higher than the French average (10 ha). Real levels of contamination could be even higher because volunteers participating in such a study were certainly more aware of prevention and more attentive to their working conditions.

To measure dermal contamination, we used the patch method, one of the two sampling strategies recommended by the OECD (OECD 2002), commonly chosen for assessing dermal exposure in fruit growing (Wolfe et al. 1975; Davis et al. 1983; de Cock et al. 1998b; Moon et al. 2013), and previously experimented in vine-growing within the PESTEXPO program (Baldi et al. 2006). This choice was made in relation to work conditions with perennial crops. As most workers are used to working with their forearms and lower legs bare during summer tasks (30% of the observations in this study), the whole-body sampling method with cotton coveralls would have been very uncomfortable for workers due to the harsh working conditions (temperatures over 30 °C in 34% of the observation days in this study). Although this method has sometimes been criticized because of the possibility of non-uniform deposits, the number and size of the patches (11 × 100 cm^2^ corresponding to 1100 cm^2^) in our study offset this limitation. Moreover, this method allowed us to estimate real dermal contamination as it took account of the usual clothes and PPE worn by the worker. The glove sampling method was preferred over handwashing when possible, as it did not modify skin permeation from one task to another on the same day. However, the glove method might have overestimated exposure since gloves have better absorption and retention capacity than the skin (Davis et al. 1983; Fenske 1990; Fenske et al. 1999). In addition, gloves were considered too uncomfortable for workers in 29 observations (wet foliage, incompatibility with the gloves usually worn by worker) and were replaced by handwashing.

In general, exposure values were higher for captan than for dithianon. This could be explained by the higher quantity of captan handled and sprayed, as the recommended dose is 1.44 kg of captan per hectare versus 0.35 kg of dithianon per hectare. In addition, most re-entry observations were during thinning days, which took place during summer when captan was more frequently applied than dithianon. Apple thinners were also more exposed because of their bare forearms and lower legs, and the significant contact between these body parts and the foliage.

Although all observations took place beyond the French legal timeframe of 48 h, the present study suggests that some re-entry tasks are more contaminant than treatments, especially thinning and anti-hail net opening. Levels of contamination in harvesters were also close to those of operators. The duration of tasks was shorter for treatment than for re-entry and harvesting, but when contamination was measured by the unit of time, dermal exposure of re-entry workers remained equal to or higher than that of operators. Nevertheless, though not negligible, dermal contamination per hour in harvesters was lower than in operators. Although the last treatments took place several weeks before harvest, our results suggest an important presence of pesticides residues on leaves or fruits at harvest. It raises questions about the effective duration of the pesticides on the crop, the pre-harvest interval and the long-term effects of the exposure of worker. Lemarchand et al. (2016) found a twofold increased risk for prostate cancer associated with pesticide use and in people harvesting fruits. An elevated risk of allergic asthma and lung squamous cell carcinoma was also associated with fruit–growing tasks (Baldi et al. 2014b; Boulanger et al. 2017).

As published data on exposure in apple growing are scarce, our results cannot be easily compared. In addition, few studies have investigated the exposure of both re-entry workers and operators in non-controlled conditions. To our knowledge, only one study, in the Netherlands in the early 1990s, had methods and objectives comparable to ours (de Cock et al. 1998b); it found that operators were more contaminated than orchard workers, which is not in line with our results. In the Dutch study, average durations of activities were similar to our observations (2 h for captan application and 6.5 h for re-entry tasks). However, Dutch operators observed in 1990–1992 wore PPE less frequently (only 34% wore gloves during mixing) and only half of them had a cabin on their tractors, while these determinants are associated with contamination (de Cock et al. 1998a). In addition, they used conventional air-blast sprayer (60%) like the trailed sprayers observed in our study, but also cross-current sprayers (37%) that we never observed. Quantities of captan handled during mixing/loading were not indicated. These differences could explain our different conclusions.

During treatment days, contamination varied between phases, with mixing/loading generally contributing the most to the full-day contamination, a result already found in some previous studies (Abbott et al. 1987; Fenske et al. 1987; Lebailly et al. 2009). Our results also suggest a high level of exposure during the equipment cleaning operations. Indeed, the median dermal contamination during cleaning was twofold higher compared to those of mixing and spraying for both active ingredients. However, differences between cleaning and the other phases were not statistically significant because few cleaning tasks were observed (*N* = 12). In our study, even though workers wore gloves during cleaning, few of them wore a coverall and 75% of them had bare forearms. This could be explained by lower risk perception, as operators were cleaning with water and were not directly handling the active ingredient. They may also have lowered their vigilance levels at the end of the day. In our study, a large majority of tractors had a four-side closed cabin, filtered and air-conditioned. However, this does not fully preclude contamination inside the cabin. Indeed, it is recommended to change the filter every year, but 5 operators ignored the date of the last change and 3 mentioned that the last change was over one year previously. In addition, the airtightness of the cabin is questionable, because it has a hole for routing the power cables to the back of the cabin. In late-model cabins, the gasket in this hole might be efficient, but the airtightness in older tractors may be questionable. Finally, even though the cabin theoretically has to be closed throughout the spraying task, reasons for opening the door and getting off the tractor were observed several times: manipulating nozzles or the rinsing tank, mechanical problems or external events. As inhalation contributes very little to daily exposure, these interventions during application may be the main source of contamination inside the cabin: after touching contaminated surfaces, operators may then contaminate the wheel and sticks; particles present in the ambient air could also be deposited in the cabin when it is open.

We observed the most common re-entry tasks performed in the orchards. Some specific tasks occurring very rarely, like plantation, were not assessed in this study. Few observation days were dedicated to pruning and bending because these operations are usually accomplished at the same time as thinning. Most of the farms involved were part of farmers’ cooperatives and did not perform apple packaging, observed only twice. Winter pruning was observed just once: we chose to observe potentially more contaminant tasks during the growing season, as winter pruning is performed a long time after the last treatments.

Differences in contamination were observed according to re-entry task. Thinning and anti-hail net opening resulted in the highest contamination, whereas lower levels were measured during anti-hail net closing. Previous studies are in line with ours, showing exposure during thinning (Wolfe et al. 1975; Davis et al. 1983; de Cock et al. 1998b; Fenske et al. 2003). However, to our knowledge, our study is the first to report exposures of workers performing anti-hail net management tasks. The anti-hail net is commonly employed to protect fruit-tree hedges and its opening cannot take place before the pollination, which occurs during the spraying season. Our results encourage to further explore the exposure of the workers performing this poorly known task.

In all tasks performed except anti-hail net opening, workers’ hands were the most contaminated body part, as expected and as observed in other studies. The forearms were also very exposed, especially during harvesting and thinning tasks, as they were directly in contact with foliage and thus dislodgeable residues (Belsey et al. 2011; Kasiotis et al. 2017). In our study, the contribution of the head to dermal exposure was also high in thinning and the highest in net opening. As all net openers and many apple thinners were observed working on a mobile platform (about 2 m high), this could be linked to the frequent contact between head and net. Further analyses will investigate this hypothesis with the help of an ergonomic approach and the analysis of dislodgeable pesticides residues on the surfaces of the potential sources of contamination (e.g., leaves, fruits, anti-hail net).

## Conclusions

This study highlights the importance of taking indirect exposures into account during re-entry work in apple growing, especially as re-entry tasks involve many seasonal workers and women who do not perform pesticide applications. As more days are spent performing re-entry tasks than treatments, pesticide exposure of workers during re-entry tasks could contribute significantly to their potential exposure during one working year (de Cock et al. 1998b). These results encourage to take a greater interest in re-entry workers in the agricultural cohorts for epidemiological studies on the effects of pesticides exposure. To understand the variability between tasks and individuals, it is necessary to identify key determinants of exposure by the analysis of field data and to study potential sources of contamination. These further analyses will be presented in further papers to explain levels of exposure in application, re-entry and harvest tasks. The help of an ergonomic approach can also provide a different light on our data. The results of this study will also help building prevention messages. Because academic studies on pesticide exposure in apple growing are scarce, especially on re-entry tasks and harvesting, our results could also help to improve the exposure models developed for the registration process.

Our results could be extrapolated to other pesticides and should contribute to estimates of the potential pesticide exposure of a worker over one working year, considering all days of treatment or re-entry and harvest days. As indirect exposures could also occur on days of regular work on the farm, we would perform further studies to assess pesticide exposure on these days without treatment, re-entry or harvest tasks.

## Data Availability

Interested persons can contact the corresponding author directly for questions relating the data.
